# Effects of sesamin on primary human synovial fibroblasts and SW982 cell line induced by tumor necrosis factor-alpha as a synovitis-like model

**DOI:** 10.1186/s12906-017-2035-2

**Published:** 2017-12-13

**Authors:** Manatsanan Khansai, Thanyaluck Phitak, Jeerawan Klangjorhor, Sasimol Udomrak, Kanda Fanhchaksai, Peraphan Pothacharoen, Prachya Kongtawelert

**Affiliations:** Thailand Excellence Center for Tissue Engineering and Stem Cells, Department of Biochemistry, Faculty of Medicine, Chiang Mai University, Chiang Mai, Thailand

**Keywords:** Sesamin, SW982, Primary human synovial fibroblast, TNF-α, Pro-inflammatory cytokines, Rheumatoid arthritis

## Abstract

**Background:**

Rheumatoid arthritis (RA) is an autoimmune disease that causes chronic synovitis, cartilage degradation and bone deformities. Synovitis is the term for inflammation of the synovial membrane, an early stage of RA. The pathogenesis of the disease occurs through cytokine induction. The major cytokine that increases the severity of RA is TNF-α. Thus, inhibition of the TNF-α cascade is an effective way to diminish the progression of the disease. We are interested in investigating the difference between primary human synovial fibroblast (hSF) cells and SW982 as synovitis models induced by TNF-α and in monitoring their responses to sesamin as an anti-inflammatory phytochemical.

**Method:**

The designed experiments were performed in hSF cells or the SW982 cell line treated with 10 ng/ml TNF-α with or without 0.25, 0.5 or 1 μM sesamin. Subsequently, pro-inflammatory cytokine genes and proteins were measured in parallel with a study of associated signalling transduction involved in inflammatory processes, including NF-κB and MAPK pathways.

**Results:**

The results demonstrated that although hSF and SW982 cells responded to TNF-α induction in the same fashion, they reacted at different levels. TNF-α could induce IL-6, IL-8 and IL-1β in both cell types, but the levels in SW982 cells were much higher than in hSF cells. This characteristic was due to the different induction of MAPKs in each cell type. Both cell types reacted to sesamin in almost the same fashion. However, hSF cells were more sensitive to sesamin than SW982 cells in terms of the anti-RA effect.

**Conclusions:**

The responses of TNF-α-induced hSF and SW982 were different at the signal transduction level. However, the two cell types showed almost the same reaction to sesamin treatment in terms of the end point of the response.

**Electronic supplementary material:**

The online version of this article (10.1186/s12906-017-2035-2) contains supplementary material, which is available to authorized users.

## Background

Rheumatoid arthritis (RA) is an autoimmune disease related to chronic joint inflammation [1]. The origin of the disease remains a mystery; however, the immune system is known to mediate the progression of diseased joints in RA [2]. The progression of RA begins with the inflammation of the synovial membrane around the affected joint (synovitis) caused by the continual immune response of many types of immune cells [3]. Therefore, the affected joint is surrounded by abundant cytokines and chemokines produced by several immune cell types. The dominant cytokine that plays a critical role in RA is tumor necrosis factor-alpha (TNF-α) [4, 5].

Previous studies demonstrated the multiple roles of TNF-α in RA progression. Remarkably, TNF-α can induce the production of inflammatory cytokines and chemokines such as IL-1β, IL-6, IL-8 and itself in synovial fibroblasts, which can increase the severity of the disease [6, 7]. TNF-α is a potent cytokine that mediates diverse effects in various cell types [8]. It is chiefly produced by monocytes and macrophages but also by B cells, T cells and fibroblasts [8]. The best-known function of TNF-α is as a mediator involved in inflammatory processes that cause RA progression [4, 5, 9]. Consequently, the accumulation of these pro-inflammatory cytokines in joints with RA can also stimulate the production of degrading enzymes, causing severe cartilage destruction [10]. TNF-α stimulation also causes nuclear factor-κB (NF-κB) and mitogen-activated protein kinase (MAPK) to play dominant roles in the progression of RA [6, 11].

The NF-κB signalling pathway has long been characterized as a pro-inflammatory signalling pathway, and the activation of NF-κB is caused by pro-inflammatory cytokines such as IL-1 and TNF-α [12]. TNF-α triggers NF-κB signalling via the TNF-α receptor located on the cell membrane. Consequently, the activation of an IκB kinase (IKK) is initiated. The IKK activation stimulates the phosphorylation of IκB at specific amino-terminal serine residues. This phosphorylation is followed by ubiquitination and degradation of the phosphorylated-IκB by the proteasome, which in turn causes the release of NF-κB dimers (p50/65) from the cytoplasmic NF-κB–IκB complex and allows them to translocate to the cell nucleus. Thereby, NF-κB binds to NF-κB enhancer elements of target genes that turn on the gene expression of pro-inflammatory cytokines, chemokines, growth factors, adhesion molecules and inducible enzymes such as cyclooxygenase-2 (COX-2) and inducible nitric oxide synthase (iNOS) [13].

In MAPK signalling, p38 MAPK (p38 mitogen-activated protein kinase), ERKs (extracellular signal–regulated kinases) and SAPK/JNK (stress-activated protein kinase/c-Jun NH (2)-terminal kinase) are involved in the TNF-α induction pathway [14–16]. The p38 MAPK pathway has been reported to be involved in the TNF-α-induced inflammatory response in synovial fibroblasts [6]. The activation of p38 MAPK allows the production of pro-inflammatory cytokines including IL-1β, TNF-α and IL-6 [6, 17]. ERKs have been reported to be activated by IL-1, TNF-α and fibroblast growth factor (FGF) in mononuclear cell infiltrates and synovial fibroblasts in synovial tissue from RA patients [18]. As ERKs are known to participate in the regulation of IL-6 and TNF-α production, there is evidence suggesting a possible role of ERKs in joint damage associated with pro-inflammatory cytokines [18]. Additionally, ERK signalling could also play a role in RA by promoting pannus formation in the affected joint [18]. The role of the SAPK/JNK MAPK signalling cascade in RA is driven by modulating the cellular responses of various pro-inflammatory cytokines, including NF-κB activation, MMP gene expression and cell survival and apoptosis [19]. Thus, this event affects the progression of RA.

The use of human cells was established to study the mechanism of RA and possible therapeutic approaches [20]. Thus, primary human fibroblast-like synoviocytes isolated from RA patients have been used to study the effects of a variety of drugs and phytochemicals [21–23]. However, there are some difficulties in using RA-derived synovial fibroblasts. They have a limited replicable lifespan and eventually enter a state of senescence, they produce a broad range of results due to the individual responses of each patient sample, and it is difficult to routinely obtain RA-derived synovial tissue samples [24, 25]. Thus, researchers have tried to use cell lines instead of primary synovial cells from patients. The best-known model used to study synovitis in RA is a human synovial sarcoma cell line (SW982) [24, 25]. The SW982 cell line has been used to examine the effects of anti-inflammatory drugs such as dexamethasone and fluvastatin in in vitro experiments [24, 25]. However, the SW982 cell line still has certain properties that are of concern for its use as an alternative cell line instead of primary human synovial fibroblast (hSF) cells from RA patients [25]. Specifically, SW982 has a self-renewal ability that is different from the behaviour of normal or RA synovial fibroblasts [26].

Sesamin is a major active compound found in sesame seeds (*Sesamum indicum* Linn.) [27]. It shows the interesting property of being associated with anti-inflammatory effects in many studies [27–29]. Previous studies showed that diets supplemented with sesamin decreased the serum levels of IL-1β and IL-6 in mice after lipopolysaccharide (LPS) exposure [27]. Other data suggested that sesamin has the ability to suppress the NF-κB and p38 MAPK pathways, which are the major pathways that control cytokine production in LPS-induced inflammation in murine microglia cells [30]. Sesamin also efficiently relieves pathological progression in the model of IL-1β-induced osteoarthritis (OA) [29]. Moreover, our previous study demonstrated a protective effect of sesamin against a cartilage degradation model induced by TNF-α and OSM [31]. On the strength of this evidence, it is possible that sesamin also inhibits cytokine production in inflammatory processes in synovitis caused by RA progression.

In this study, we aim to investigate and clarify the responses of different RA models, TNF-α-induced Primary human synovial fibroblast (hSF) cells and the SW982 cell line, to sesamin treatment. The effects of sesamin on both models were examined by investigation of the pro-inflammatory gene expression including IL-1β, IL-6, IL-8, and TNF-α. The release of IL-6 and IL-8 was reported as pro-inflammatory cytokine production. Furthermore, the NF-kB and MAPK signalling pathway were studied as signalling pathways that regulate the inflammatory processes.

## Methods

### Chemicals

Chemicals and supplements were purchased from the following suppliers: cell culture supplements such as Dulbecco’s Modified Eagle’s Medium (DMEM), penicillin, streptomycin and 0.25% Trypsin EDTA were purchased from Life Technologies (Burlington, Ontario, Canada). Recombinant Human TNF-α was purchased from Peprotech (Rocky Hill, USA). Sesamin was extracted from sesame seeds (*Sesamum indicum* Linn.) that were harvested from Lampang Province of Thailand. The voucher specimens (BKF No. 138181) were submitted to the National Park, Wildlife and Plant Conservation Department, Ministry of Natural Resources and Environment, Bangkok, Thailand. The processes were administered by Assoc. Prof. Dr. Wilart Poompimol. The chemical structure of the sesamin extract was analysed by NMR/MS (MW 354.35) as described in our previous publication. The RNA Isolation Kit was obtained from GE Health Science (New York, USA). The Tetro cDNA Synthesis Kit was purchased from BIOLINE (Taunton, USA). SsoFast™ EvaGreen Supermix was purchased from Bio-Rad (Bio-Rad Laboratories (Singapore) Pte. Ltd.). A real-time PCR machine was purchased from Bio-Rad (Bio-Rad Laboratories (Singapore) Pte. Ltd.). The MILLIPLEX MAP Human Cytokine, Chemokine and Immuno Cell Multiplex Assays Kit was obtained from Merck Millipore (Merck KGaA, Darmstadt, Germany). Anti-human β-actin, anti-IκB, anti-phospho IκB, anti-p65, anti-phospho p65, anti-SAPK/JNK, anti-phospho SAPK/JNK, anti-p38, anti-phospho p38, anti-p44/42, anti-phospho p44/42, goat anti-rabbit IgG conjugated HRP and horse anti-mouse IgG conjugated HRP were obtained from Cell Signaling Technology (Danvers, MA, USA). Bradford reagent was obtained from Bio-Rad (Bio-Rad Laboratories (Singapore) Pte. Ltd.). Nitrocellulose membranes were purchased from Amersham (Hybond-C Super, Amersham Pharmacia Biotech). A semi-dry blot machine was purchased from Bio-Rad (Bio-Rad Laboratories (Singapore) Pte. Ltd.). The SuperSignal West Femto Maximum Sensitivity Substrate Kit and Restore™ plus Western blot stripping buffer were purchased from Thermo Scientific (Thermo Fisher, Waltham, Massachusetts, USA). A gel documentary system was purchased from Bio-Rad (Bio-Rad Laboratories (Singapore) Pte. Ltd.).

### Primary human synovial fibroblast (hSF) isolation, culture and treatment

Primary human synovial fibroblast (hSF) cells were isolated by a method previously described for obtaining tissue-derived fibroblast-like synovial cells [32]. Synovial tissue was obtained from knee joints of patients undergoing joint removal surgery (the ethics approval code was ORT-11-09-16A-14.). The synovial tissue was minced in a tissue culture dish with Dulbecco’s Modified Eagle Medium (DMEM) containing 200 units/ml penicillin, 200 mg/ml streptomycin and 50 μg/ml gentamicin and supplemented with 20% foetal calf serum. The minced tissue was maintained in a humidified incubator with 5% CO_2_ at 37 °C. After 4 days of culture, the tissue was taken out, and the adhered cells were washed with phosphate buffered saline (PBS). Cells were maintained in the growth medium at 5% CO_2_ and 37 °C. The cells from passages 3 through 6 were used in this experiment.

### SW982 synovial sarcoma cell line culture and treatment

SW982 was obtained from ATCC® number HTB-93 and was authenticated by DiagCor Bioscience Incorporation Limited using the Promega Powerplex® 18D system and analysed using a ABI 3130 Genetic Analyzer. The cells were cultured in a sealed 25 ml T–culture flask with Leibovitz-15 (L-15) medium containing 200 units/ml penicillin, 200 mg/ml streptomycin and 50 μg/ml gentamicin and supplemented with 10% foetal calf serum in a 37 °C humidified incubator.

### Cytotoxicity test

SW982 or hSF cells were seeded at concentrations of 1 × 10^4^ cells/well in 96-well culture plates for 24 h. After 24 h, the cells were treated with TNF-α (0.625–20 ng/ml) or sesamin (0.125–5 μg/ml) alone or co-treated with TNF-α (10 ng/ml) and sesamin (0.25, 0.5, and 1 μM) for 48 h. Cell viability was measured by the MTT assay. Absorbance was measured at 540 nm. The percentage of cell survival was obtained using the formula below:

$$ \%\mathrm{cell}\  \mathrm{survival}=\frac{100\times {\mathrm{OD}}_{540}\mathrm{treated}\  \mathrm{cells}}{{\mathrm{OD}}_{540}\mathrm{control}\  \mathrm{cells}} $$

### Real-time polymerase chain reaction (Real-Time PCR) assay

SW982 or hSF cells were cultured in a 25 ml T–culture flask (sealed flasks were used for SW982) until they reached 80% confluence. The cells were cultured in serum-free medium (L-15 for SW982, DMEM for hSF) for 24 h. The effects of sesamin on inflammation were investigated by treatment with or without 10 ng/ml human recombinant TNF-α and 0.25, 0.5 and 1 μM of sesamin for 4 h after fasting for analysis. The total RNA was isolated by using RNA Isolation Reagent (GE Health Science) according to the manufacturer’s instructions. One microgram of total RNA was used for reverse transcription to produce cDNA using the Tetro cDNA Synthesis Kit. The transcribed cDNAs were mixed with SsoFast™ EvaGreen Supermix and the level of mRNA expression was evaluated using a Chromo4 real-time PCR detection system. The human-specific primer sequences were as follows: GAPDH, F: 5’GAAGGTGAAGGTCGGAGTC3’ and R: 5’GAAGATGGTGATGGGATTTC3’; IL-1β, F: 5’AAACAGATGAAGTGCTCCTTCCAGG3’ and R: 5’TGGAGAACACCACTTGTTGCTCCA3’; IL-6, F: 5’GGTACATCCTCGACGGCATCT3’ and R: 5’GTGCCTCTTTGCTGCTTTCAC3’; IL-8, F: 5’CTCTCTTGGCAGCCTTCC3’ and R: 5’CTCAATCACTCTCAGTTCTTTG3’; and TNF-α, F: 5’CCCCAGGGACCTCTCTCTAATC3’ and R: 5’GGTTTGCTACAACATGGGCTACA3’. The data were normalized with respect to the constitutive gene GAPDH and analysed quantitatively using the 2^-ΔΔCT^ formula [33].

### Immunological multiplex assays

SW982 or hSF cells were cultured in 25 ml T–culture flasks (sealed flasks were used for SW982) until they reached 80% confluence. The cells were maintained in serum-free L-15 medium (for SW982) or DMEM (for hSF) for 24 h prior to treatment with or without 10 ng/ml human recombinant TNF-α and 0.25, 0.5 or 1 μM of sesamin for 48 h [34]. After treatment, the cell culture supernatant was collected and its IL-6, IL-8 and IL-1β levels measured by MILLIPLEX MAP Human Cytokine, Chemokine and Immuno Cell Multiplex Assays.

### Western blotting

Both cell types were cultured in 25 ml T–culture flasks (sealed flasks were used for SW982) until they reached 80% confluence. The culture medium was then replaced with serum-free L-15 medium (for SW982) or DMEM (for hSF) for 24 h prior to pre-treatment with serum-free L-15 or DMEM containing 0.25, 0.5 or 1 μM of sesamin for 2 h. Next, human recombinant TNF-α (final concentration = 10 ng/ml) was added to each flask, and the cell lysate was collected at several time points (0, 5, 10, 15 and 30 min). Cell lysate was harvested by scraping with 200 μl ice-cold RIPA buffer containing protease inhibitor and phosphatase inhibitor. The protein concentrations of the samples were determined by using the Bradford protein assay and then calculated. The protein concentration was adjusted to equal amounts before loading on SDS-PAGE (13% separating gel, 5% stacking gel). The protein samples were electrophoretically separated and transferred to nitrocellulose membranes by a semi-dry blot system. The membranes were then blocked with 5% (*W*/*V*) non-fat dry milk in Tris-buffered saline with 0.1% Tween 20 (TBS-T) for 1 h at room temperature. Then, the membranes were washed with TBS-T prior to being incubated overnight at 4 °C with primary antibodies against human β-actin or IκB, phospho-IκB, p65 and phospho-p65 to prepare samples for studying NF-κB signal transduction (1:1000 in TBS-T). The samples prepared for studying MAPK signal transduction (1:1000 in TBS-T) were incubated with SAPK/JNK, phospho-SAPK/JNK, p38, phospho-p38, p44/42 and phospho-p44/42 antibodies overnight at 4 °C. Next, the membranes were washed 5 times for 5 min with TBT-T prior to being incubated with secondary antibodies conjugated with horseradish peroxidase (1:1000 in TBS-T) for 1 h at room temperature. The resulting blots were washed 5 times for 5 min with TBS-T before visualization using the SuperSignal West Femto Maximum Sensitivity Substrate Kit to obtain an enhanced chemiluminescence signal. The visualized results were recorded using a gel documentary system.

### Statistical analysis

The data were expressed as the mean ± SEM from triplicate samples of three independent experiments. One-way ANOVA was used to assess the differences between conditions. Significance was defined as *p* < 0.05.

## Results

### hSF and SW982 cells showed different responses in terms of TNF-α-induced pro-inflammatory cytokines mRNA expression, and sesamin showed anti-inflammatory effects by suppressing pro-inflammatory cytokine and chemokine gene expression in both models

**Fig. 1:**
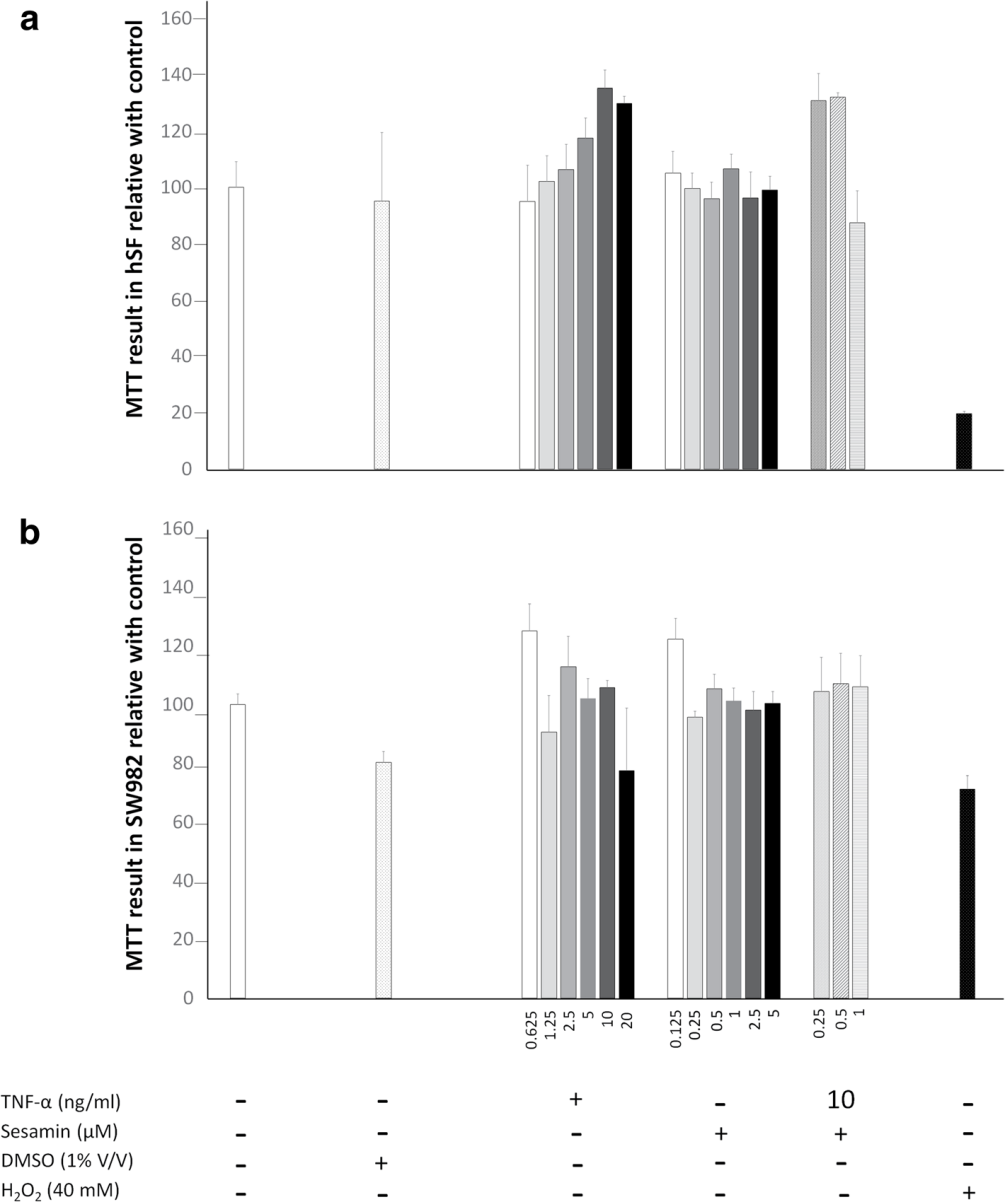
Cell viability testing of hSF and SW982 cells. Cell viability testing was performed by the MTT assay as described in the *Materials and Methods* section. **a** Cell viability results for hSF cells under TNF-α alone, sesamin alone or combinations of TNF-α and sesamin. **b** Cell viability results for SW982 cells under TNF-α alone, sesamin alone or combinations of TNF-α and sesamin. Values are presented as the mean ± SEM (*n* = 3)

We optimized the concentrations of TNF-α and sesamin that were used in the experiments, and cell viability was determined using the MTT assay, as described previously. SW982 cells and hSF cells were exposed to TNF-α concentrations ranging from 0.625 to 20 ng/ml (2-fold dilution), sesamin concentrations ranging from 0.125 to 5 μM or a combination of 10 ng/ml TNF-α with 0.25, 0.5 or 1 μM of sesamin for 48 h. Cells treated with 40 mM H_2_O_2_ were used as a toxic control. The results suggested that cell viability was not affected compared to that of the control when hSF or SW982 cells were treated with TNF-α, sesamin or both (Fig. 1). We also confirmed the nontoxicity of all treatments that were used in this study by LDH assay, the result is presented in Additional file 1.

**Fig. 2:**
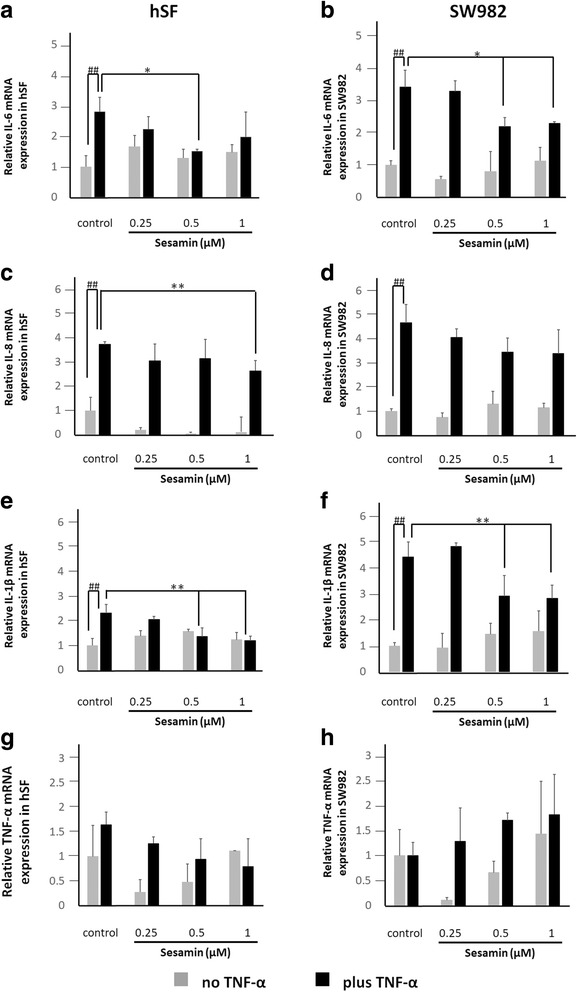
Fold induction of IL-6, IL-8, IL-1β and TNF-α mRNA expression in each cell type when treated with TNF-α or sesamin or co-treated with TNF-α and various concentrations of sesamin compared to respective controls. hSF or SW982 cells were treated and the gene expression profiles measured as described in the *Materials and Methods* section. **a, b** Fold induction of IL-6 gene expression in hSF and SW982 cells, respectively. **c, d** Fold induction of IL-8 gene expression in hSF and SW982 cells, respectively. **e, f** Fold induction of IL-1β gene expression in hSF and SW982 cells, respectively. **g, h** Fold induction of TNF-α gene expression in hSF and SW982 cells, respectively. Values are presented as the mean ± SEM (*n* = 3). #, * = *p* < 0.05; ##, ** = < 0.01 versus normal control (#) or TNF-α treatment (*) by one-way ANOVA

The changes in pro-inflammatory cytokine and chemokine gene expression, including IL-1β, IL-6, TNF-α and IL-8, in hSF and SW982 cells after treatment with 10 ng/ml TNF-α were investigated using real-time PCR. When both cell types were exposed to TNF-α, hSF exhibited significantly increased levels of IL-6, IL-8, IL-1β and TNF-α mRNA expression compared to those of its own control (Fig. 2). However, SW982 exhibited significantly increased levels of IL-6, IL-8, and IL-1β but not TNF-α mRNA expression (Fig. 2g).

The mRNA expression experiment demonstrated that sesamin could suppress the mRNA expression of pro-inflammatory cytokines that were induced by TNF-α (Fig. 2). Sesamin concentrations of 0.5 and 1 μM significantly reduced IL-6, IL-8 (only at 1 μM) and IL-1β mRNA expression in hSF cells, while TNF-α mRNA expression was not decreased compared to that of the induction control (Fig. 2a, c, e, and g). In SW982, sesamin could significantly reduce IL-6 and IL-1β gene expression similar to in hSF but could not reduce IL-8 and TNF-α expression (Fig. 2b, d, f, and h). However, sesamin alone could not affect all involved pro-inflammatory cytokine and chemokine gene expression levels in both cell types (Fig. 2).

### The levels of pro-inflammatory cytokine and chemokine production induced by TNF-α in hSF and SW982 were different, and sesamin showed an anti-inflammatory effect by suppressing pro-inflammatory cytokine and chemokine production in both models

**Fig. 3:**
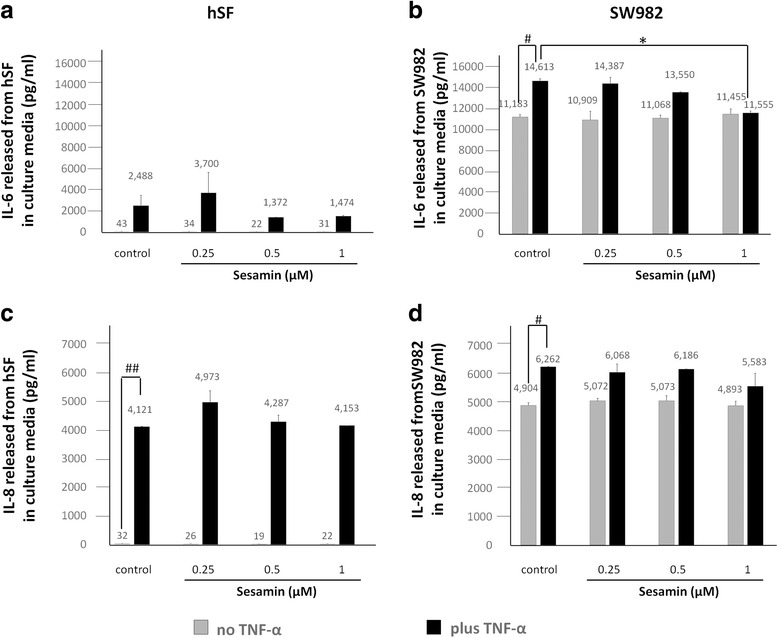
Levels of IL-6 and IL-8 released in the culture medium of hSF and SW982 cells. Both cell types were treated and assayed as described above. The levels of IL-6 and IL-8 released in the cultured medium were analysed by Luminex assays. **a, b** Levels of IL-6 production from hSF and SW982 cells, respectively. **c, d** Amounts of IL-8 released from hSF and SW982 cells, respectively. Values are expressed as the mean ± SEM (*n* = 3). #, * =  *p* < 0.05; ##, ** =  *p* < 0.01 versus normal control (#) or TNF-α treatment (*) by one-way ANOVA

The levels of secretion of pro-inflammatory cytokines and chemokines including IL-1β, IL-6 and IL-8 from hSF and SW982 were determined using MILLIPLEX MAP Human Cytokine, Chemokine and Immuno Cell Multiplex Assays. Even though the levels of IL-1β production were measured for both cell types, the value of this cytokine was not detected using this technique because the concentration did not reach the minimum level for this test. The level of TNF-α released was not determined due to the presence of added TNF-α in the treatment condition. We determined a baseline level of IL-6 and IL-8 release from hSF and SW982 cells (Fig. 3). The results were consistent with the gene expression results. After 48 h of cultivation, TNF-α treatment increased the IL-6 and IL-8 production compared to that of the control, as expected (Fig. 3). Moreover, when exposed to TNF-α, hSF and SW982 cells showed very different increases in the release of both IL-6 and IL-8 into the culture medium (Fig. 3). After induction, the IL-6 and IL-8 production levels of hSF cells were increased by approximately 60- and 100-fold vs. the control, while SW982 responded by increasing both IL-6 and IL-8 release by only approximately 1.3-fold vs. the control (Fig. 3).

At the protein production level, the results showed a significant reduction in IL-6 release in the presence of 1 μM sesamin in the co-treatment conditions for SW982 only (Fig. 3b). At other concentrations, the presence of sesamin in co-treatment conditions demonstrated a slightly decreased effect in both cell types (Fig. 3). Additionally, treatment with sesamin alone could not affect all the relevant pro-inflammatory cytokines and chemokines produced in both models (Fig. 3). Interestingly, although hSF and SW982 cells exhibited different levels of cytokine and chemokine response to TNF-α activation, they responded to sesamin treatment in almost the same fashion.

### TNF-α activated a different signalling pathway in hSF from that in SW982, and sesamin suppressed the TNF-α-induced inflammatory response by interfering with MAPK signal transduction

**Fig. 4:**
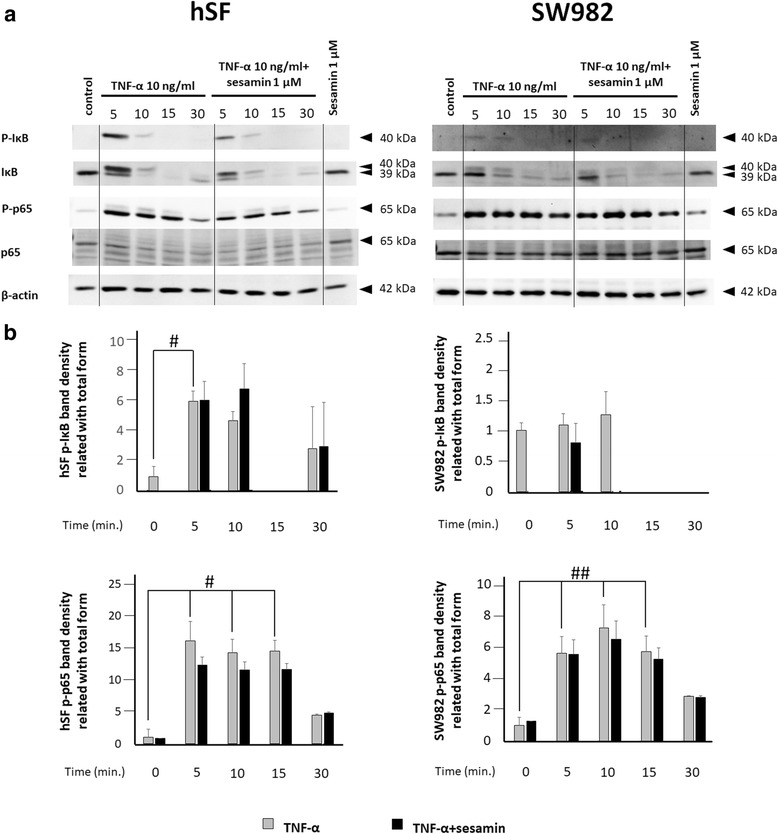
Western blot analysis of NF-κB signal transduction in hSF and SW982 cells. The NF-κB signalling was observed l at 0, 5, 10, 15 and 30 min after the addition of TNF-α 10 ng/m to hSF or SW982 cells pre-treated with 1 μM sesamin as described in the *Materials and Methods* section. **a** Western blot results of NF-κB signalling in hSF and SW982 under TNF-α-induced conditions with or without sesamin treatment. **b** Graphs of phosphorylation band densities of IκB and p65 relative to the total form of each cell type. Values are presented as the mean ± SEM (n = 3). #, * =  *p* < 0.05; ##, ** =  *p* < 0.01 versus control 0 min (#) or TNF-α treatment at various time points (*) by one-way ANOVA

At the molecular level, we determined the NF-κB and MAPK signal transduction of TNF-α in both cell types by Western blot analysis. To investigate the NF-κB signalling pathway, we monitored the changes in phosphorylation of IκB and p65 at various time points. The Western blot results showed that TNF-α induced pro-inflammatory cytokine and chemokine via NF-κB signalling in both cell types (Fig. 4a). The phosphorylation of IκB was significantly initiated at 5 min after both cell types were exposed to TNF-α (Figs. 4a, b). Furthermore, when comparing the phosphorylation strength of IκB using the value of band density relative to its own band density (total form), we found that in hSF cells, the phosphorylation strength of IκB was significantly increased at 5 min, while in SW982 cells, the phosphorylation strength of IκB was slightly increased at 5 and 10 min compared to the strength during the non-stimulation stage (Fig. 4b). Significant phosphorylation of p65 in hSF and SW982 cells occurred in approximately 5 to 15 min (Fig. 4b). These data indicate the same type of NF-κB activation in response to TNF-α induction in both cell types.

Treatment with 1 μM sesamin in parallel with exposure to 10 ng/ml TNF-α demonstrated that sesamin had no effect on the phosphorylation of IκB in hSF cells, while in SW982 cells, the phosphorylation of IκB showed a slight decrease in induction at 5 and 10 min (Fig. 4). The phosphorylation of p65 in hSF and SW982 cells also showed similar results (Fig. 4b). The phosphorylation of p65 was not affected by the presence of sesamin (Fig. 4b).

**Fig. 5:**
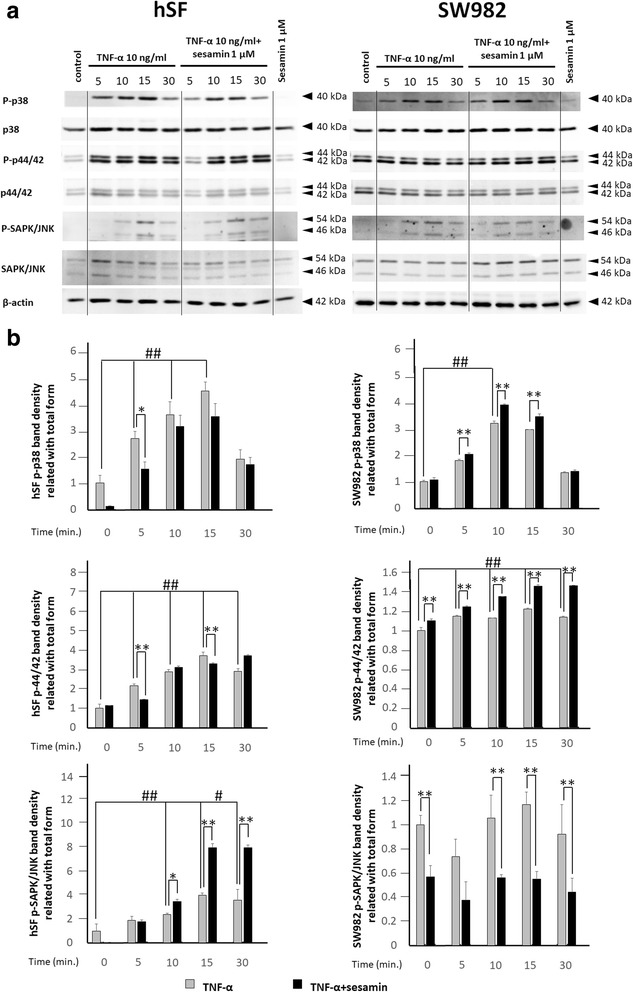
Western blot analysis of MAPK signal transduction in hSF and SW982 cells. MAPK signalling was observed at 0, 5, 10, 15 and 30 min after the addition of TNF-α 10 ng/ml to hSF or SW982 cells pre-treated with 1 μM sesamin as described in the *Materials and Methods* section. **a** Western blot results of MAPK signalling in hSF (left panel) and SW982 (right panel) cells under TNF-α-induced conditions with or without sesamin treatment. **b** Graphs of phosphorylation band densities of p38, p44/42 (ERK) and SAPK/JNK relative to the total form in hSF (left panel) and SW982 (right panel) cells. Values are presented as the mean ± SEM (n = 3). #, * =  *p* < 0.05; ##, ** =  *p* < 0.01 versus control 0 min (#) or TNF-α treatment at various time points (*) by one-way ANOVA

To study the triggering of the MAPK signalling pathway by TNF-α in hSF and SW982 cells, we investigated the changes in the phosphorylation of p38, p44/42 (ERK1/2) and SAPK/JNK, as in the NF-κB study (Fig. 5). It is noteworthy that this monitoring demonstrated a clear difference in MAPK induction by TNF-α between hSF and SW982 cells. The phosphorylation of p38 after induction in hSF demonstrated a significant increase from 5 to 15 min, while the phosphorylation of p44/42 exhibited a significant increase throughout the experiment (Fig. 5). The results of SAPK/JNK showed a significant increase in phosphorylation at 10 to 30 min after cytokine induction. Meanwhile, TNF-α-induced SW982 showed effects on p38 and p44/42 but not SAPK/JNK (Fig. 5). These data indicate different forms of MAPK activation in response to TNF-α activation in the two cell types.

In the investigation of MAPK signalling in hSF cells, the presence of sesamin in the induction system caused a significant reduction in phosphorylated p38 and p44/42 (especially p44/42) at 5 and 15 min of induction (Fig. 5b). For SAPK/JNK signal transduction, the data showed different effects (Fig. 5b). Sesamin continuously increased the phosphorylation of SAPK/JNK (Fig. 5b). These data indicated that sesamin could slightly reduce the activation of p38 and p44/42 ERK induced by TNF-α in hSF cells and shift to activation of the SAPK/JNK signalling pathway. Interestingly, the data showed a reverse effect on SW982 cells (Fig. 5b). Sesamin increased the activation of p38 and p44/42 ERK but decreased the activation of SAPK/JNK in the SW982 cell line (Fig. 5b).

## Discussion

Rheumatoid arthritis (RA) is a chronic disease that is manifested by joint inflammation and leads to irreversible joint deformation in the late stages. The basis of this disease remains unclear. However, many reports demonstrate an association between an abnormal immune system and the functions of connective cells around the joint lesion. The main secretion produced by immune cells that plays a key role in RA is TNF-α [9]. Many studies have evaluated the relationship of TNF-α with RA in several models [35, 36]. Animal models are the most commonly used in RA research [35, 36]. Although animal models can yield an overall understanding of RA pathogenesis, these models also have serious limitations. Due to discrepancies between human arthritis and animal models of arthritis, some responses are different [20]. Importantly, although many drugs have shown great potency in animal models, this advantage has not been borne out in clinical trials [20, 36]. Relatedly, the key point of RA progression is associated with the inflammation of the synovial membrane around the RA joint [3]. Thus, in vitro models that use human cells were developed to study the mechanism of RA and possible therapeutic approaches [20]. Therefore, primary human fibroblast-like synoviocytes from RA patients have been used to screen and study the unique effects of many drugs and phytochemicals [21–23]. Despite the advantages of primary human fibroblast-like synoviocytes, they present certain inconveniences; for instance, primary mammalian cells have a limited replicable lifespan and ultimately enter a state of senescence in which they remain metabolically active but fail to proliferate. The lack of reproducibility as a result of the individual response of each patient sample and the need to routinely acquire RA-derived synovial tissue samples make such studies difficult [24, 25]. Thus, researchers have tried to establish cell models instead of using primary synovial cells from patients. The candidate cell line SW982, obtained from the American Tissue Culture Collection, has been used in many models of RA [24, 25]. The SW982 cell line was established by A. Leibovitz in 1974 at the Scott and White Clinic, Temple, Texas. These cells were isolated from a surgical specimen of a biphasic synovial sarcoma from a 25-year-old female Caucasian patient [37]. Although the SW982 cell line has been widely used in research examining the mechanism of RA, the exact evidence and scientific rationale that supports the use of this cell line as an alternative to primary synovial fibroblasts are still unclear [24, 25].

In our experiment, we used naïve synovial fibroblast (hSF) cells isolated from joint removal patients without OA or RA. The reason we used naïve cells is that such tissue specimens are more accessible than RA-derived synovial tissue. In this study, it was found that sesamin treatment did not affect non-stimulant inflammation in either SW982 or hSF cells. These responses appeared at both the gene and protein expression levels. These phenomena suggest that sesamin has no effect on the ordinary cell activity of either the SW982 cell line or hSF cells.

In the resting stage, hSF cells expressed a low level of inflammatory cytokine production, while SW982 cells released a very high level of inflammatory cytokines. These results confirm the difference in using SW982 and hSF in an RA model for inflammation conditions. We used TNF-α as a stimulant to induce acute inflammation in both hSF and SW982 to mimic the inflammation in RA progression. We found that both hSF and SW982 exhibited a similar response. In acute inflammation conditions, both hSF and SW982 cells responded by increasing their IL-6, IL-8, IL-1β and TNF-α mRNA levels. Similar results were also observed in the protein production levels. However, the degree of response was different. The reaction of hSF was stronger than that of SW982 due to the unfamiliarity of the cytokine attack. When sesamin was present in the induction system, we found that sesamin could significantly reduce the mRNA expression of IL-6, IL-8 and IL-1β but not TNF-α in hSF cells. In SW982 cells, we found significantly decreased levels of only IL-6 and IL-1β mRNA expression. However, a significant reduction in cytokine release in the presence of sesamin was only observed for IL-6 produced by SW982 cells at only the highest concentration of sesamin. These incompatible results could be explained by the different time periods that we used in each experiment. When investigating mRNA expression, we incubated the cells with TNF-α and sesamin for 4 h, but when measuring cytokine release, we cultured the cells with the inducer and sesamin for 48 h. The different time periods were used to obtain appropriate time points. For cells cultivated with TNF-α, mRNA first reaches its maximum level at 4 h (according to the kinetics of immune response) [38, 39]. However, it was necessary to incubate the cells with TNF-α for 48 h for an appropriate accumulation of cytokines to be released [34]. In fact, during the 48-h period, many immune-related genes were also activated, and many kinds of cytokines and chemokines were produced and degraded to maintain homeostasis [9]. These phenomena also affected the amounts of cytokines that we measured. Moreover, the difference in cell passages that we used also affected the cellular response, which is why hSF showed a broader range of cytokines released than SW982.

We monitored the signalling transduction triggered by TNF-α in both cell models. Both NF-κB and MAPK signalling was examined. We found that both hSF and SW982 cells showed partial phosphorylation of p65 in the resting stage. This result was supported by the baseline protein level of inflammatory cytokines that we found in the previous experiments. Moreover, in the first 5 min after exposure to TNF-α, hSF cells increased the activation of phosphorylated IκB and p65 to reach full activation. However, this reaction decreased with the time passed. Meanwhile, SW982 cells responded similarly to the hSF cells, but p65 activation was retained longer in SW982 cells. The NF-κB transcription factor is well known as a critical regulator of inflammation in RA. Thus, the blocking of the NF-κB signalling pathway may be a potential therapeutic approach for RA. However, our study showed that sesamin could not reduce the activation of NF-κB in either cell type, although the phosphorylation of IκB differed slightly different in the two models. Another signalling pathway we monitored was MAPK. We investigated the activation of p38, p44/42 (ERK1/2) and SAPK/JNK by examining their phosphorylation. According to our results, TNF-α could induce all three members of the MAPK pathway in hSF cells. However, in SW982 cells, TNF-α activated only the phosphorylation of p38 and p44/42 but not SAPK/JNK. SW982 exhibited a high degree of p44/42 phosphorylation even in the resting stage. Additionally, the phosphorylation of p44/42 or ERK1/2 MAPK is involved in the regulation of various processes including cell cycle progression, transcription, cell proliferation, cell differentiation, cell metabolism, cell adhesion, cell migration and cell survival [40]. In this case, the fully activated phosphorylation of p44/42 in SW982 may be related to the immortal activity that is found in cancer cells. Thus, this difference in phosphorylation is a difference in the properties of cell lines and of primary cells. Our results indicated that in a TNF-α-induced system, sesamin decreased the phosphorylation of p38 and p44/42 but increased the phosphorylation of SAPK/JNK in hSF cells. In contrast, sesamin significantly increased the phosphorylation of p38 and p44/42 but decreased the phosphorylation of SAPK/JNK in SW982 cells. These results demonstrated the different responses of hSF and SW982 to sesamin, an anti-inflammatory phytochemical. However, overall, both cell types responded to sesamin in almost the same fashion, except that hSF cells seemed to be more sensitive to sesamin than SW982 cells.

Our study demonstrated the advantages and disadvantages of using hSF and SW982 as a model of RA. Remarkably, hSF and SW982 had distinct inflammatory process characteristics in terms of signal transduction and gene expression changes involving cytokine production. Many RA studies have chosen SW982 as cell model because of a high proliferation rate that appears similar to pannus formation in the severe stage of RA. However, the use of SW982 could be a concern because of the immortal activity of this cell. The utility of hSF cells as an inflammation model for RA study may be improved by pre-incubating cells with an appropriate concentration of TNF-α for a suitable time period before the addition of any phytochemicals. The development of this method will exclude the unwanted properties of SW982 as an inflammatory cell model. The investigation of the effects of the phytochemical sesamin on both established models showed that they both respond to sesamin in the same fashion. However, the levels of mRNA and protein expression and the activation of intracellular signalling were different. Thus, both established models could be used as drug screening models for RA treatment. Nevertheless, the correct underlying mechanism must still be investigated.

## Conclusion

In this study, different mechanisms that control the inflammatory response of TNF-α-induced hSF and SW982 cells were identified. However, both models could be used to investigate the anti-RA properties of phytochemical agents. They showed almost the same response to sesamin at the gene and protein expression levels. However, the signalling transduction response to sesamin treatment in the two cell types was different. Therefore, the correct underlying intracellular signalling should be of concern when using SW982 as a model.

## Additional file

Additional file 1: LDH released from hSF and SW982 cells. Cell viability testing was performed by LDH measurement in culture medium. **a.** LDH released from hSF cells under TNF-α alone, sesamin alone or combination of TNF-α and sesamin. **b.** LDH released from SW982 cells under TNF-α alone, sesamin alone or combination of TNF-α and sesamin. Values are presented as mean ± SEM (*n* = 3). (PNG 135 kb)

## Abbreviations

cDNA: Complementary deoxyribonucleic acid
COX-2: Cyclooxygenase-2
DMEM: Dulbecco’s Modified Eagle Medium
F: Forward
HRP: Horseradish peroxidase
hSF: Primary human synovial fibroblast
IKK: Inhibitor of NF-κB kinases
IL-1β: Interleukin-1 beta
IL-6: Interleukin-6
IL-8: Interleukin-8
iNOS: Inducible nitric oxide synthase
IκB: Inhibitor of NF-κB
L-15: Leibovitz-15 medium
LPS: Lipopolysaccharide
mRNA: Messenger ribonucleic acid
MW: Molecular weight
NF-κB: Nuclear factor-κB
NMR/MS: Nuclear magnetic resonance spectroscopy and mass spectrometry
OA: Osteoarthritis
OSM: Oncostatin M
p38 MAPK: p38 mitogen-activated protein kinase
p44/42 MAPK (ERK1/2): Extracellular signal–regulated kinases (ERKs) or classical mitogen-activated protein kinase
PBS: Phosphate buffered saline
p-IκB: Phosphorylated inhibitor of NF-κB
p-p38 MAPK: Phosphorylated p38 mitogen-activated protein kinase
p-p44/42 MAPK (Erk1/2): Phosphorylated extracellular signal–regulated kinases 1/2 (ERK1/2)
p-p65: Phosphorylated p65
p-SAPK/JNK: Phosphorylated stress-activated protein kinase/c-Jun NH(2)-terminal kinase
R: Reverse
RA: Rheumatoid arthritis
SAPK/JNK: Stress-activated protein kinase/c-Jun NH(2)-terminal kinase
SDS-PAGE: Sodium dodecyl sulfate-polyacrylamide gel electrophoresis
SW982: Human synovial sarcoma cell line
TBS: Tris-buffered saline
TBS-T: Tris-buffered saline with 0.1% Tween 20
TNF-α: Tumor necrosis factor-alpha
β-actin: Beta-actin
