# Supplementary figures and images for: Effects of sesamin on primary human synovial fibroblasts and SW982 cell line induced by tumor necrosis factor-alpha as a synovitis-like model
Source: BMC Complement Altern Med. 2017 Dec 13;17:532. doi: 10.1186/s12906-017-2035-2 (PMC5729244; doi:10.1186/s12906-017-2035-2)

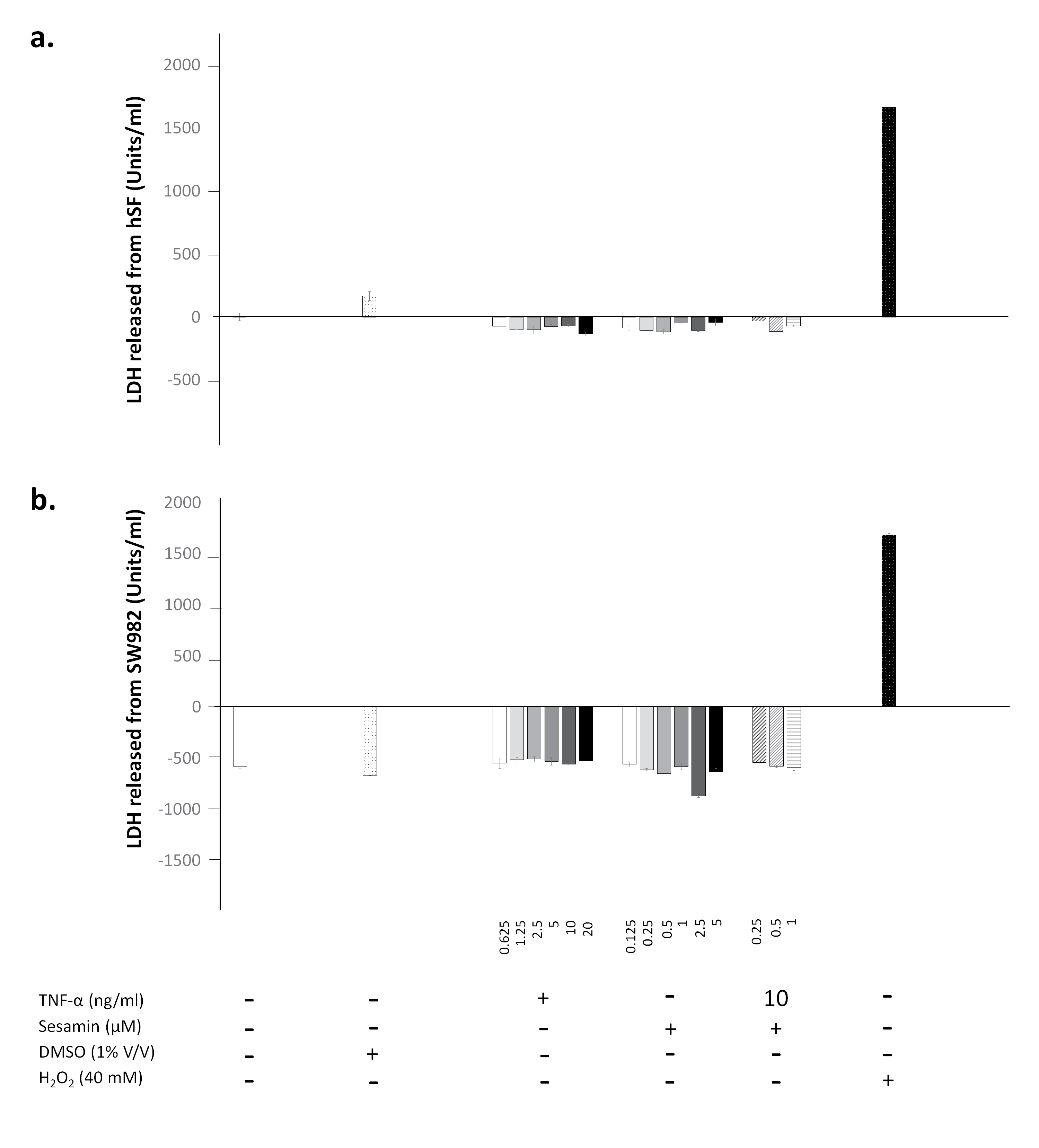

Supplement: Additional file 1: — LDH released from hSF and SW982 cells. Cell viability testing was performed by LDH measurement in culture medium. a. LDH released from hSF cells under TNF-α alone, sesamin alone or combination of TNF-α and sesamin. b. LDH released from SW982 cells under TNF-α alone, sesamin alone or combination of TNF-α and sesamin. Values are presented as mean ± SEM (n = 3). (PNG 135 kb) [file 12906_2017_2035_MOESM1_ESM.png]
